# The Effect of Medical Marijuana Laws on Crime: Evidence from State Panel Data, 1990-2006

**DOI:** 10.1371/journal.pone.0092816

**Published:** 2014-03-26

**Authors:** Robert G. Morris, Michael TenEyck, J. C. Barnes, Tomislav V. Kovandzic

**Affiliations:** Program in Criminology, University of Texas at Dallas, Richardson, Texas, United States of America; Tulane University School of Public Health and Tropical Medicine, United States of America

## Abstract

**Background:**

Debate has surrounded the legalization of marijuana for medical purposes for decades. Some have argued medical marijuana legalization (MML) poses a threat to public health and safety, perhaps also affecting crime rates. In recent years, some U.S. states have legalized marijuana for medical purposes, reigniting political and public interest in the impact of marijuana legalization on a range of outcomes.

**Methods:**

Relying on U.S. state panel data, we analyzed the association between state MML and state crime rates for all Part I offenses collected by the FBI.

**Findings:**

Results did not indicate a crime exacerbating effect of MML on any of the Part I offenses. Alternatively, state MML *may* be correlated with a reduction in homicide and assault rates, net of other covariates.

**Conclusions:**

These findings run counter to arguments suggesting the legalization of marijuana for medical purposes poses a danger to public health in terms of exposure to violent crime and property crimes.

## Introduction

The social ramifications of marijuana legalization have been hotly debated for at least four decades [1]. Despite a long history of marijuana use for medical purposes, policymakers and in some instances, the scientific community, have been quick to note the potential problematic social outcomes of marijuana legalization [2]. In spite of these political discussions, medical marijuana legalization (MML) has occurred in 20 states and the District of Columbia (between 1996 and the writing of this paper) and its recreational use has now been legalized in Colorado and Washington [3]. An interest in the ramifications of these laws has led to an increase in scholarly activity on the topic [4], [5]. The issue addressed in this article is whether MML has the effect of increasing crime. While there are many mechanisms by which MML might affect crime rates, the most obvious is by increasing the number of marijuana users, which may lead to a broader social acceptance of drug using behaviors and drug users [6]. To the extent that marijuana use serves as a “gateway” to harder drugs such as cocaine and heroin, MML could lead to long-term increases in crime as an ever-growing number of illicit drug users engage in serious predatory crimes to support their habits (but see [7]). But even if MML does not lead to a rise in marijuana use (especially among youth), the laws could still stimulate crime as newly opened medical marijuana dispensaries provide criminals with a highly attractive target with their repository of high quality marijuana and customers carrying large amounts of cash (but see [8]). As a member of the California Chiefs of Police Association stated, “A disturbing and continuing trend is the increasing number of home invasion robberies and associated violence resulting in the victimization of those cultivating and possessing marijuana … [D]ispensaries also continue to be targeted based upon the availability of larger quantities of drugs and cash” (see http://californiapolicechiefs.org/wp-content/uploads/2012/02/July_September_2010_Final.pdf). Though anecdotal evidence abounds to support both theses, and a few single-jurisdiction and cross-sectional studies have examined the MML-crime link (e.g., [9]), no single analysis has assessed the overall consequences of medical marijuana laws on crime rates across the United States. This study seeks to inform the debate by providing a comprehensive evaluation of the effects of state MML on state crime rates.

### The Positive Correlation between Marijuana Use and Criminal Behavior

Though the gateway hypothesis applies to the progression of drug-using behaviors, there remains the possibility that marijuana use leads to delinquent or criminal behavior via a similar mechanism. A number of studies have specifically examined the relationship between marijuana use and crime [10], [11], [12], [13], [14]. Early studies compared the amount of crimes committed by juveniles whose urine tested positive for marijuana upon entering a detention center and those committed by individuals who tested negative for marijuana. Dembo and associates [15], [16], for instance, found that youths who tested positive for marijuana had a significantly higher number of referrals to juvenile court for nondrug felonies than those testing negative for marijuana use.

Arseneault and colleagues [17] examined the relationship between marijuana dependence and the risk for violence in a sample of New Zealand adolescents. The authors controlled for gender, socioeconomic status, and many other concurrent disorders and concluded that marijuana dependence was related to a 280 percent increase in the odds of violence. This association was stronger than the individual effects of manic disorder, alcohol dependence, and schizophrenia. In a study using data collected from school-age adolescents in the Netherlands, those who reported marijuana use tended to report more delinquent and aggressive behaviors [18]. This relationship was significant after controlling for variables such as alcohol and tobacco use and the strength of the relationship increased with higher frequency of marijuana use. This study is noteworthy because marijuana use is decriminalized in the Netherlands, thus the relationship is unlikely to be based on the fact that marijuana users have to participate in the illegal market and are therefore at an increased risk for violence. While these studies were cross-sectional and show a correlation between current marijuana use and criminality or violent behaviors, other scholars have examined the link with longitudinal data.

Using multi-wave data, research has shown adolescents who reported marijuana use at age 15 were more likely to report violent involvement at age 19, indicating that marijuana use, particularly during adolescence may impact violent behavior in young adulthood [19]. Similarly, research has shown that frequent marijuana use during adolescence was a strong predictor of being involved in intimate partner violence [5]. Results revealed that consistent marijuana use during adolescence was related to a 108 percent increase in the likelihood of being involved in intimate partner violence in young adulthood and consistent marijuana use was associated with an 85 percent increase in the odds of being the perpetrator of intimate partner violence, independent of alcohol use.

These studies provide evidence to the notion that marijuana use is at a minimum correlated with an increase in violent or aggressive behaviors. What remains unclear is whether these findings imply a causal link between marijuana use and violence or whether the relationship is driven by an uncontrolled variable(s) (i.e., a spurious correlation). Along these lines, it could be argued that the relationship between violence and marijuana use is primarily due to its illegality and thus would not exist in an environment in which marijuana use, at least medicinally, is legalized.

### The Negative or Null Correlation between Marijuana Use and Criminal Behavior

Most researchers who have examined the relationship between marijuana use and crime report that these laws do not have an effect on violent crime [20], [21]. Green and associates [20], for instance, concluded that while marijuana use was related to an increase in drug and property crime, it was not related to an increase in violent crime. Pedersen and Skardhamar [21] also found a relationship between marijuana use and subsequent arrest, although once the authors removed all types of drug charges from the models, the relationship was no longer significant. Results revealed no evidence that marijuana use was related to an increase in later non-drug arrest, such as arrests for violent crimes. The authors argued that the association between marijuana use and crime appears to exist because of its illegality. Thus, if the possession and sale of marijuana was legal the relationship between marijuana and crime might disappear.

It has been argued that medicinal marijuana laws may increase crime because the dispensaries and grow houses provide an opportunity for property crime and violent crime to occur, such as burglary and robbery. Kepple and Freisthler [9] examined the relationship between medical marijuana dispensaries and crime and their results suggested that after controlling for a host of ecological variables, no relationship existed between medicinal marijuana dispensaries and property or violent crime. Additional research has shown that medical marijuana dispensaries may actually reduce crime within the immediate vicinity of the dispensaries [8]. This may be due to the security measures implemented by dispensary owners (i.e., having security cameras, having a doorman, and having signs requiring identification). Importantly, medical marijuana dispensaries do not appear to increase crime in their surrounding areas.

In sum, research on the relationship between medicinal marijuana and crime is mixed. Studies have shown that states allowing the use of medical marijuana have higher prevalence rates of marijuana use [13], [14], yet other studies have found that legalized medicinal marijuana does not lead to an increase in its overall use [21], [22]. Research has also suggested that marijuana use is associated with an increase in illicit drug use [23], [19] and an increase in crime [17], [19], [16]. Others, however, have revealed that marijuana is not related to additional illicit drug use [22], [7], [17] or crime [8], [20], [9], [21]. Thus, the available evidence is equivocal and in need of a rigorous evaluation of the MML-crime relationship.

## Methods

### Data & Measures

#### Dependent Variables

Data on all seven Part I offenses—homicide, rape, robbery, assault, burglary, larceny, and auto theft—for each state between 1990 and 2006 were obtained from the Federal Bureau of Investigation's Uniform Crime Reporting (UCR) Program, published as *Crime in the United States*. The data were obtained using the “data for analysis” tool on the Bureau of Justice Statistics Web site (http://www.ojp.usdoj.gov/bjs/dtd.htm). All data were gathered for each of the 50 U.S. states across the 17 year time span for a total *N* = 850. Values reflect the rate of each crime per 100,000 residents.

#### Medical Marijuana Legalization (MML)

To determine if and when MML occurred within a state, we searched the official legislative website of each US state. Between 1990 and 2006, the following 11 states legalized marijuana for medical use, with the year the law was passed in parentheses: Alaska (1998), California (1996), Colorado (2000), Hawaii (2000), Maine (1999), Montana (2004), Nevada (2000), Oregon (1998), Rhode Island (2006), Vermont (2004), and Washington (1998). We also ran models based on MML “legislation-effective year” rather than “legislation-passed year” and found no substantive differences in the results. The MML effective dates were also gathered from each State's official legislative website. Only 2 states (Connecticut and Colorado) had an MML effective year different than “passed” year, both being only a 1-year difference. While there are many options in modeling the effects of MML adoption on crime, we opted to use a post-law trend variable. The trend variable represents the number of years the law has been in effect with a value of zero for all years before the law was passed, a value of 1 for the year the law was passed, and a value of 1+*k*, where *k*  =  number of years after the initial passage of the law, for all subsequent years. Unlike the traditional “dummy variable” approach (i.e., 0  =  no MML law, 1  =  MML law), which posits a once-and-for-all impact on crime, the post-law trend variable captures any changes in the linear trend of crime that may be observed over time. If opponents of MML are correct that the laws lead to increased marijuana use by teenagers, many of whom are likely to continue illicit hard drug use throughout their adulthood, one might expect a gradual increase in crime over time. Such an effect would be best captured by the post-law trend variable.

#### Sociodemographic Control Variables

Sociodemographic variables were included in the analysis to aid in controlling for a vast array of other time-varying influences that might be potential confounding factors over the study period. These variables, and their sources, have been described previously [24]. Specifically, they include each state's percent of the civilian labor force unemployed; the total employment rate; percent of the population living below the poverty line; real per-capita income (divided by the Consumer Price Index); the proportion of residents aged 15–24; the proportion of residents aged 25–34, the proportion of residents aged 35–44 years; the per-capita rate of beer consumption [25]; the proportion of residents with at least a bachelor's degree; and the percent of the state's population that lived in a metropolitan area. State-level unemployment data were obtained from the Bureau of Labor Statistics website (www.bls.gov/sae/home). Data on poverty were acquired via the Bureau of the Census website (www.census.gov/hhes/www/poverty). Personal income and real welfare payments data were taken from the Bureau of Economic Analysis website (www.bea.doc.gov/bea/regional/reis). The age variables were obtained directly from the U.S. Bureau of the Census. Data on beer consumption were taken from the Beer Institute website (www.beerinstitute.org). The percent of the population with college degrees or higher and the percent of the population living in a metropolitan area are linear interpolations of decennial census data, as reported in various editions of the *Statistical Abstracts of the United States*.

Additional measures included the number of prison inmates per 100,000 residents and the number of police officers per 100,000 residents. The number of prisoners was measured as the number of prisoners sentenced to more than a year in custody as of December 31 per 100,000 residents and was obtained from the Bureau of Justice Statistic's website (www.ojp.usdoj.gov/bjs). Data on the total number of police, including civilians, were taken from the Public Employment series prepared by the Bureau of the Census. Louisiana and Mississippi were missing information on this variable for the year 2006, therefore reducing the usable case count by two units. Substantive results were identical when values for this year were imputed with values from the previous year. Summary statistics for these explanatory variables are presented in Table 1.

**Table 1:** Summary Statistics.

	Mean	SD
***Dependent Variables (prior to log transformation)***		
Homicide Rate	5.778	3.347
Rape Rate	36.774	13.212
Robbery Rate	130.346	91.687
Assault Rate	303.573	161.996
Burglary Rate	845.706	304.654
Larceny Rate	2,727.552	687.953
Auto Theft Rate	406.504	208.103
***Independent Variable***		
Medical Marijuana Law (Post-law Trend)	.393	1.489
***Sociodemographic control variables***		
Unemployment rate	5.162	1.393
Employment rate	58,568.89	5,043.444
Poverty rate	12.442	3.638
Real per-capita income	5.193	.844
Proportion persons ages 15 to 24	.142	.011
Proportion persons ages 25 to 34	.145	.017
Proportion persons ages 35 to 44	.156	.011
Beer shipments (31-gallon barrels) per 100k	73,670.89	12,003.72
Percent persons with college degree	23.897	4.903
Percent persons residing in metropolitan area	67.654	20.636
Prisoners per 100k	343.072	144.897
Police officers per 100k	278.473	48.917

Note: Descriptive statistics are for the 1990–2006 period. The data sources are noted in the text.

### Analysis Plan

To identify the effect of MML on crime, we use a fixed-effects panel design, exploiting the within state variation introduced by the passage of MML in 11 states over the 17 year observation period. The design allows for the assessment of whether states adopting MML experienced changes in the trend of crime by analyzing within state changes in crime rates over time and comparing those changes to the crime rate trends among states that did not pass an MML law. To carry out this analysis, we estimate fixed-effects ordinary least squares regression models, where the natural log of each crime rate variable (i.e., homicide, rape, robbery, assault, burglary, larceny, and auto theft) is the dependent variable. This model directly accounts for dynamic factors that cause crime to vary from state to state, as well as those stable unmeasured factors that differ between states [26], [27]. In addition, we also include “year fixed-effects,” which capture any national influences on crime that are not captured in any of the time-varying explanatory variables. Robust standard errors are clustered at the state level to avoid biased standard errors due to the non-independence of data points over time [28]. Thus, the fixed effects models can be expressed algebraically following the convention set forth by Wooldridge [27] as: 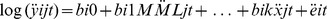



where:

the subscripts *i*, *j*, and *t* are used to identify the crime rate variable being used as the dependent variable, the 50 states, and time (1990–2006), respectively;

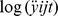
  =  the time-demeaned (see [27]) logged crime rate outcome variable;

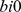
  =  the crime-specific constant term;

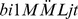
  =  the time-demeaned crime-specific average impact of MML on crime rates;

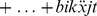
  =  the time-demeaned crime-specific effect of the various control variables, including year dummies, a linear trend variable, and state fixed effects;
and, 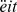 =  the time-demeaned crime-specific error term.

It is important to note that fixed-effects models are not without limitations. While they are well suited to address the issue at hand and account for unobserved time-invariant factors, they are always vulnerable to time-varying factors that are not accounted for that differ between states with MML and those without. However, we have accounted for the bulk of factors that have been shown associated with state crime rates and our models explain a considerable amount of variation in each outcome. It is also important to acknowledge that fixed-effects models do not account for temporal ordering for time-varying predictors within a given observation period. For example, it is unknown whether states adopted MML after experiencing lower crime rates in a given year(s), however, this is unlikely to be an issue here since policy response to crime rates tend to take time and we account for this via operationalization of MML as an additive effect.

## Results

### Primary Findings

**Figure 1:**
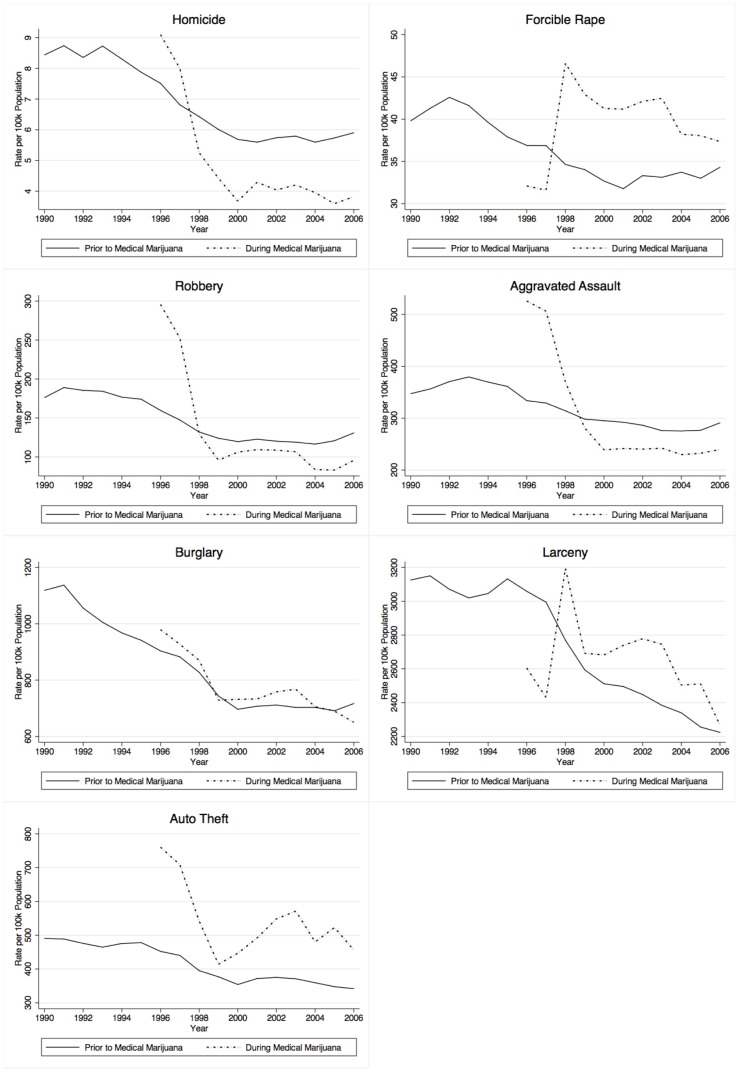
Mean State Crime Rates as a Function of Year, by Medical Marijuana Law (MML). NOTE: Crime rates for states mandating MML after 1996 remained in the “Prior to Medical Marijuana” line until transition to MML.

Before consulting the results from the fixed effects regression models, a series of unconditioned crime rates for each offense type were generated and are presented in Figure 1. Note that two crime rate trends are presented in each panel. One trend—the solid line—shows the crime rate, by year, for states that had *not* passed an MML law. Thus, states that eventually did pass an MML law contribute to the solid line up until the year that they passed the MML law. As expected from the overall crime trend during this time period, the solid line reveals that all states experienced a reduction in each of the seven crimes from 1990 to 2006. Important to note is the trend revealed by the dashed line, which shows the crime rate trends for states *after* passing an MML law. With one exception—forcible rape—states passing MML laws experienced reductions in crime and the rate of reduction appears to be steeper for states passing MML laws as compared to others for several crimes such as homicide, robbery, and aggravated assault. The raw number of homicides, robberies, and aggravated assaults also appear to be lower for states passing MML as compared to other states, especially from 1998–2006. These preliminary results suggest MML may have a crime-reducing effect, but recall that these are unconditional averages, meaning that the impact of the covariates and other factors related to time series trends have not been accounted for in these figures.

The results of the fixed effects analyses are presented in Table 2. It is important to note that a Hausman test was carried out to determine whether the fixed effects model was preferable over the random effects model; the latter model is more parsimonious and, thus, should be preferred when results do not systematically differ across the two approaches. The results of the Hausman tests (with year fixed effects omitted for both equations because they are inestimable in the random effects model) suggested that the fixed effects model was preferred in each of the seven analyses. For reference, the Hausman χ^2^ values were 302.61, 23.64, 102.50, 414.94, 58.87, 34.18, and 31.28 for homicide, rape, robbery, assault, burglary, larceny, and auto theft, respectively.

**Table 2:** The Impact of Medical Marijuana Laws on Crime Rates.

Variable	Homicide	Rape	Robbery	Assault	Burglary	Larceny	Auto Theft
Medical Marijuana Law (MML)	−0.024***	−0.005	−0.016	−0.024*	−0.004	−0.002	0.026
	(0.007)	(0.009)	(0.010)	(0.013)	(0.007)	(0.004)	(0.016)
Unemployment rate	0.031**	−0.001	0.039**	−0.021	0.022**	0.005	0.036**
	(0.012)	(0.014)	(0.015)	(0.022)	(0.011)	(0.009)	(0.017)
Employment rate	1.325	3.672***	3.637**	4.249***	0.420	−0.584	−0.069
	(1.277)	(1.156)	(1.536)	(1.383)	(0.943)	(0.747)	(1.715)
Poverty rate	−0.008**	0.006	0.001	0.001	−0.004	−0.002	−0.007*
	(0.003)	(0.004)	(0.005)	(0.005)	(0.003)	(0.002)	(0.004)
Per-capita income	−0.013	−0.226***	−0.148**	−0.173*	−0.194***	−0.099***	−0.137
	(0.057)	(0.067)	(0.072)	(0.100)	(0.048)	(0.036)	(0.102)
Proportion aged 15 to 24	3.528	−0.279	−3.591	−3.245	0.676	−0.266	5.279
	(2.447)	(1.681)	(3.371)	(2.961)	(1.696)	(1.422)	(3.509)
Proportion aged 25 to 34	−4.250**	−0.202	−3.478	−7.492**	5.150***	2.729	11.352***
	(1.884)	(2.038)	(2.920)	(3.112)	(1.904)	(1.712)	(2.609)
Proportion aged 35 to 44	−1.393	−3.083	−4.008	−13.777***	−1.940	0.193	−3.558
	(2.041)	(2.319)	(3.366)	(4.654)	(1.928)	(1.489)	(4.075)
Beer consumption	0.903**	0.504*	1.261***	0.436	0.857***	0.762***	1.376**
	(0.399)	(0.283)	(0.442)	(0.576)	(0.291)	(0.280)	(0.580)
Percent college degree	−0.004	0.016	−0.032**	−0.012	−0.001	0.005	−0.018
	(0.011)	(0.010)	(0.012)	(0.017)	(0.007)	(0.007)	(0.013)
Percent metropolitan	0.015**	0.022**	0.004	0.004	−0.006	−0.005	−0.009
	(0.007)	(0.008)	(0.009)	(0.015)	(0.008)	(0.006)	(0.014)
Prisoners per 100k	−45.675	−20.410	−33.918	41.979	−7.186	9.724	−56.412
	(33.964)	(22.442)	(35.013)	(30.046)	(26.127)	(18.575)	(48.726)
Police officers per 100k	−0.001	0.000	−0.002	−0.001*	−0.000	0.001	−0.001
	(0.001)	(0.001)	(0.001)	(0.001)	(0.001)	(0.001)	(0.002)
*R* ^2^	.50	.46	.58	.44	.83	.75	.44

Robust standard errors in parentheses.

*** p<0.01, ** p<0.05, * p<0.1

Note: State fixed-effects and year fixed-effects are included in all estimates but are not shown in the table. The following variables were divided by 100000 in order to produce coefficients that did not require scientific notation to interpret: Employment rate, Beer consumption, and Prisoners per 100k.

The key results gleaned from the fixed effects analyses are presented in row 1 of Table 2, which reveals the impact of the MML trend variable on crime rates, while controlling for the other time-varying explanatory variables. Two findings worth noting emerged from the different fixed effects regression analyses. First, the impact of MML on crime was negative or not statistically significant in all but one of the models, suggesting the passage of MML *may* have a dampening effect on certain crimes. The second key finding was that the coefficients capturing the impact of MML on homicide and assault were the only two that emerged as statistically significant. Specifically, the results indicate approximately a 2.4 percent reduction in homicide and assault, respectively, for each additional year the law is in effect. Because log-linear models were estimated, the coefficient must be transformed according to the following formula to generate percentage changes in crime for a one-unit increase in MML: *e* ^(*b*-1)*100^ [27]. However, it is important to note that the finding for homicide was less variable (i.e., a lower standard error) as compared to assault. One might argue a Bonferroni correction is necessary given the exploratory nature of the study and the multiple models that were analyzed. Once a Bonferroni correction was carried out (i.e., α/7), only the effect of MML on homicide remained statistically significant (.05/7 = .007). Perhaps the most important finding in Table 2 is the lack of evidence of any increase in robbery or burglary, which are the type of crimes one might expect to gradually increase over time if the MML-crime thesis was correct. Thus, in the end, MML was not found to have a crime enhancing effect for any of the crime types analyzed.

### Sensitivity Analyses

The fixed effects models presented above were subjected to a range of sensitivity tests to determine whether the findings were robust to alternative model specifications. First, and as previously noted, data for the two missing cases were imputed using matched case replacement for Louisiana and Mississippi. Importantly, substantive results were identical when this strategy was carried out. A second sensitivity analysis explored the possibility that the effect of MML on crime rates was non-linear. No evidence emerged to support the hypothesis that MML has a non-linear effect on crime rate trends. Third, a related issue concerns whether the MML effect has *both* a trend effect (shown above) *and* a one-time shock effect. We considered this issue by including the MML trend variable (discussed above) along with a dummy variable coded 0 for years when no MML law was present (by state) and coded 1 in years when an MML law had been passed. The findings were practically identical to those shown above: the MML trend variable was negatively related to homicide (*b* = −.02, *p*<.10) and assault (*b* = −.02, *p*<.10). A fourth sensitivity analysis re-estimated the original models (shown above), by weighting each state proportional to its population size. When these weighted fixed effects models were estimated, the substantive findings were somewhat different than those presented above. Specifically, the effect of MML on homicide rates was no longer statistically significant (*b* = −.01, *p* = .30), MML negatively predicted robbery rates (*b* = −.02, *p*<.10), MML negatively predicted assault rates (*b* = −.03, *p*<.01), and MML *positively* predicted auto theft rates (*b* = .03, *p*<.05). While it is common in the crime policy literature to weight observations by resident population to correct for possible heteroskedasticity, this will be the efficient feasible GLS (generalized least squares) procedure only if the heteroskedasticity takes a particular form, i.e. variance proportional to the square of the population. In the present study, the unweighted results produce findings that are substantively consistent with the weighted results, although they differ slightly quantitatively. The most likely explanation for this discrepancy is that the weighted results are driven by a few large population states. For this reason, we present the unweighted results as the main results and the weighted results as part of our numerous robustness checks.

## Discussion and Conclusion

The effects of legalized medical marijuana have been passionately debated in recent years. Empirical research on the direct relationship between medical marijuana laws and crime, however, is scant and the consequences of marijuana use on crime remain unknown. Studies have shown that marijuana use was associated with higher prevalence of subsequent illicit drug use [19] and an increased risk of violence [17]. Yet, other studies have found that once additional factors were controlled for, there was no relationship between marijuana use and later serious drug use [7]. Research has also shown that marijuana use is not related to violent crime when measured at the individual-level [20]. Once drug charges are controlled for, Pedersen and Skardhamar [21] reported that the relationship between marijuana and crime was not significantly different from zero. Unfortunately, no study has examined the effect of legalized medical marijuana on state crime rates across the United States. The current study sought to fill this gap by assessing the effect of legalized medicinal marijuana on the seven Part I UCR offenses. The analysis was the first to look at multiple offenses across multiple states and time periods to explore whether MML impacts state crime rates.

The central finding gleaned from the present study was that MML is not predictive of higher crime rates and *may* be related to reductions in rates of homicide and assault. Interestingly, robbery and burglary rates were unaffected by medicinal marijuana legislation, which runs counter to the claim that dispensaries and grow houses lead to an increase in victimization due to the opportunity structures linked to the amount of drugs and cash that are present. Although, this is in line with prior research suggesting that medical marijuana dispensaries may actually reduce crime in the immediate vicinity [8].

In sum, these findings run counter to arguments suggesting the legalization of marijuana for medical purposes poses a danger to public health in terms of exposure to violent crime and property crimes. To be sure, medical marijuana laws were *not* found to have a crime exacerbating effect on any of the seven crime types. On the contrary, our findings indicated that MML precedes a reduction in homicide and assault. While it is important to remain cautious when interpreting these findings as evidence that MML *reduces* crime, these results do fall in line with recent evidence [29] and they conform to the longstanding notion that marijuana legalization may lead to a reduction in alcohol use due to individuals substituting marijuana for alcohol [see generally 29, 30]. Given the relationship between alcohol and violent crime [31], it may turn out that substituting marijuana for alcohol leads to minor reductions in violent crimes that can be detected at the state level. That said, it also remains possible that these associations are statistical artifacts (recall that only the homicide effect holds up when a Bonferroni correction is made).

Given that the current results failed to uncover a crime exacerbating effect attributable to MML, it is important to examine the findings with a critical eye. While we report no positive association between MML and any crime type, this does not *prove* MML has no effect on crime (or even that it reduces crime). It may be the case that an omitted variable, or set of variables, has confounded the associations and masked the true positive effect of MML on crime. If this were the case, such a variable would need to be something that was restricted to the states that have passed MML, it would need to have emerged in close temporal proximity to the passage of MML in all of those states (all of which had different dates of passage for the marijuana law), and it would need to be something that decreased crime to such an extent that it “masked” the true positive effect of MML (i.e., it must be something that has an opposite sign effect between MML [e.g., a positive correlation] and crime [e.g., a negative correlation]). Perhaps the more likely explanation of the current findings is that MML laws reflect behaviors and attitudes that have been established in the local communities. If these attitudes and behaviors reflect a more tolerant approach to one another's personal rights, we are unlikely to expect an increase in crime and might even anticipate a slight reduction in personal crimes.

Moreover, the present findings should also be taken in context with the nature of the data at hand. They are based on official arrest records (UCR), which do not account for crimes not reported to the police and do not account for all charges that may underlie an arrest. In any case, this longitudinal assessment of medical marijuana laws on state crime rates suggests that these laws do not appear to have any negative (i.e., crime exacerbating) impact on officially reported criminality during the years in which the laws are in effect, at least when it comes to the types of offending explored here. It is also important to keep in mind that the UCR data used here did not account for juvenile offending, which may or may not be empirically tethered to MML in some form or another; an assessment of which is beyond the scope of this study.
